# In vitro susceptibility of *Burkholderia cepacia* to ceftazidime-avibactam: A systematic review

**DOI:** 10.1016/j.nmni.2025.101601

**Published:** 2025-05-28

**Authors:** Abolfazl Rafati Zomorodi, Leila Ghanbari_Afra, Hossein Faridafshar, Seyed Nooreddin Faraji, Fatemeh Zarepour, Maryamosadat Mavaei, Mohammad Rahmanian

**Affiliations:** a Department of Bacteriology and Virology, School of Medicine, Shiraz University of Medical Sciences, Shiraz, Iran; b Instructor, Department of Medical Surgical Nursing, Faculty of Nursing, Qom University of Medical Sciences, Qom, Iran; c Department of Microbiology, School of Medicine, Kermanshah University of Medical Sciences, Kermanshah, Iran; d Student Research Committee, School of Medicine, Kermanshah University of Medical Sciences, Kermanshah, Iran; e Department of Pathology, School of Medicine, Shiraz University of Medical Sciences, Shiraz, Iran; f School of Medicine, Kashan University of Medical Sciences, Kashan, Iran; g Pharmaceutical Sciences Research Center, Health Institute, Kermanshah University of Medical Sciences, Kermanshah, Iran; h Student Research Committee, Kermanshah University of Medical Sciences, Kermanshah, Iran; i Student Research Committee, School of Medicine, Shahid Beheshti University of Medical Sciences, Tehran, Iran; j Gastroenterology and Liver Diseases Research Center, Research Institute for Gastroenterology and Liver Diseases, Shahid Beheshti University of Medical Sciences, Tehran, Iran

**Keywords:** *Burkholderia cepacia*, Ceftazidime/avibactam, Cystic fibrosis, Respiratory tract infections, Systematic review

## Abstract

**Background:**

*Burkholderia cepacia* complex (BCC) is a group of multi-drug resistant (MDR) pathogens that are challenging to treat, especially in cystic fibrosis (CF) patients. Ceftazidime-avibactam is a promising antibiotic combination for treating BCC infections, but its efficacy requires further in vitro evaluation.

**Methods:**

This systematic review was conducted following the PRISMA guidelines to assess the in vitro susceptibility of BCC strains to ceftazidime-avibactam. We systematically searched PubMed, Web of Science, Scopus, and Embase databases. Inclusion criteria required original articles reporting on BCC susceptibility to ceftazidime-avibactam using standard antimicrobial susceptibility testing methods.

**Results:**

A total of 9 studies met the inclusion criteria. These studies were conducted between 2010 and 2024, with data from the USA, France, Germany, Belgium, and other countries. The studies used various methods, including agar dilution, broth microdilution, and disc diffusion. The minimum inhibitory concentration (MIC) range for ceftazidime-avibactam was found to vary, with the combination showing significantly improved susceptibility compared to ceftazidime alone.

**Conclusion:**

This systematic review demonstrates that ceftazidime-avibactam significantly enhances the susceptibility of BCC strains, supporting its potential as an effective therapeutic option for BCC infections in CF patients. Further clinical studies are needed to confirm these findings and guide treatment strategies.

## 1 Introduction

Global public health faces a critical challenge in antimicrobial resistance, emphasizing the need for targeted antibiotic development against gram-negative bacteria, major contributors to resistant infections. The *Burkholderia cepacia* complex (BCC), a group of Gram-negative bacteria, comprises approximately twenty pathogenic species. Effective treatment of BCC infections is impeded by their inherent resistance to commonly used antibiotics and the development of in vivo biofilms. The distinctive double membrane structure of their cell envelope, notably in BCC species, poses a significant barrier to antibiotic penetration. BCC, especially in immunocompromised individuals, is associated with persistent and multidrug-resistant infections [1,2]. The presence of BCC in cystic fibrosis (CF) patients' airways is associated with increased morbidity and mortality due to resistance to antibiotic therapy [3].

*B. cenocepacia*, a member of the BCC bacteria, induces cepacia syndrome, characterized by pneumonia and bacteremia, posing a challenge for treatment due to extensive resistance. Prolonged antibiotic cocktails are often required for eradication, lasting weeks to months [4]. Another concern is *B. multivorans*, a significant health threat to individuals with CF, demonstrating inherent resistance to multiple antibiotics, complicating treatment options [5]. The BCC and *B. gladioli* are opportunistic pathogens that most commonly infect persons with CF or compromised immune systems. Members of the *Burkholderia* genus are intrinsically multi-drug resistant (MDR), possessing both a PenA carbapenemase and an AmpC β-lactamase, rendering treatment of infections due to these species problematic [6]. BCC and *B. gladioli* produce two inducible β-lactamases, belonging to the Pen-like class A and AmpC [7].

CF patients are exhibiting an increasing incidence of recovery of other MDR non-fermenters, including *Burkholderia gladioli*, *Achromobacter*, *Cupriavidus*, *Inquilinus*, *Pandoraea*, and *Ralstonia*. The novel cephalosporin/β-lactamase inhibitor combinations, ceftolozane/tazobactam and ceftazidime/avibactam, demonstrate promising activity against a range of Gram-negative bacilli, including various *Burkholderia* species. However, the in vitro efficacy of these combinations has not been fully explored regarding interspecies variations, and direct comparisons using identical strain panels are limited [8].

Previous studies have shown that ceftazidime-avibactam exhibited substantial potency against BCC, *B. gladioli* strains and MDR strains of *P. aeruginosa* in CF patients [7,9]. Considering the essential nature of various bacterial cell envelope components without human homologues, they represent attractive targets for diverse antibiotics [10]. Overcoming the antimicrobial resistance (AMR) pandemic requires multiple interventions, including the development of novel antimicrobials for treating drug-resistant infections. The STEDI values, encompassing Spectrum, Transmission, Enablement, Diversity, and Insurance, play a crucial role in evaluating antimicrobial efficacy. The molecular mechanisms of ceftazidime, avibactam, and ceftazidime/avibactam involve targeting PBP3/Divisome, PBP2/Rod complex, and both mechanisms, respectively, leading to the disruption of cell wall synthesis [11]. This systematic study delves into the in-vitro studies regarding the susceptibility of *Burkholderia cepacia* to ceftazidime-avibactam, exploring its efficacy and implications for the treatment landscape.

## 2 Methods

We used the Preferred Reporting Items for Systematic Reviews and Meta-Analysis: the PRISMA Statement [12] to report our study.

### 2.1 Search strategy

Four main databases comprising PubMed/MEDLINE, Web of Science, Scopus, and Embase were searched for the assessment of susceptibility of *B. cepacia* spp. against ceftazidime/avibactam. The systematic search was performed between 2000 and May 2025 using the following keywords: “*Burkholderia cepacia*” OR “*B. cepacia*” OR “*Burkholderia cepacia* complex” OR “*Burkholderia* spp.” OR “gram-negative bacilli” AND “Antimicrobial susceptibility testing” OR “antimicrobial resistance patterns” OR “ceftazidime/avibactam” OR “ceftazidime drug combination” OR “ceftazidime-avibactam” OR “avibactam” OR “AVE1330A”. The search was limited to original articles published in English. In addition, manual reviews were conducted of the reference list of identified articles.

### 2.2 Inclusion and exclusion criteria

The original articles that presented data on the susceptibility of *B. cepacia* spp. against ceftazidime/avibactam were checked independently by two authors and based on title, abstract and full-text; the fourth author ARZ evaluated discrepancies. The eligible articles were included regarding: (i) original articles that reported the susceptibility of *B. cepacia* spp. against ceftazidime/avibactam; (ii) tests were carried out using standard antimicrobial susceptibility testing methods such as agar dilution method, microbroth or microbroth dilution, and Etest. Exclusion criteria were: (i) non-human samples; (ii) abstracts presented at conference; (iii) any types of review articles, (iv) and duplicate publications.

### 2.3 Data extraction

The following characteristics were extracted from selected articles by two independent researchers: the author's last name, year of publication, type of study, country, year of investigation, number of *B. cepacia* spp. isolates, MIC range (mg/L), MIC_90_ and MIC_50_, number of ceftazidime resistance isolates, number of ceftazidime/avibactam susceptible isolates, number of MDR and extensively-drug resistant (XDR) isolates, and the source of isolates.

## 3 Results

**Fig. 1:**
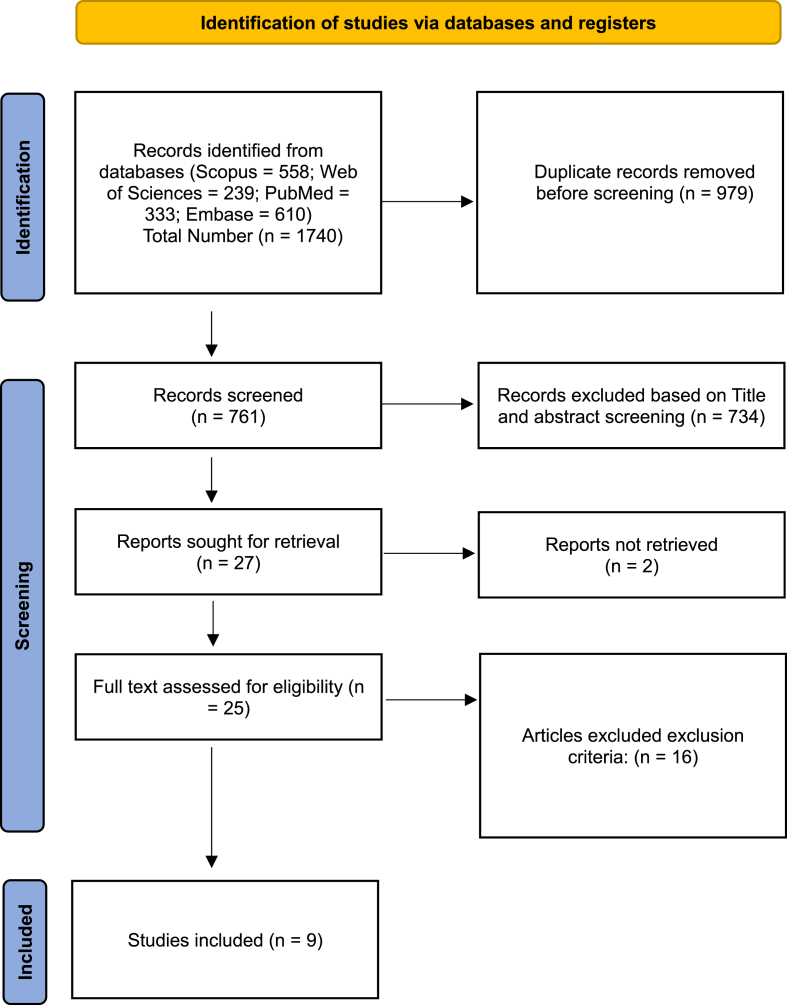
PRISMA flow diagram chart of the number of studies identified and included in systematic review.

This systematic review identified 1740 articles related to the research topic through database searches. After removing duplicates (n = 979), screening titles and abstracts (n = 734), applying exclusion criteria (n = 16), and accounting for non-retrieved articles (n = 2), a total of 9 articles were reviewed (Fig. 1).

Included articles were conducted between 2010 and 2024. The information was obtained from research conducted in the USA-Australia [13], France [14], USA-Canada [15], Belgium [16], Germany [17], Spain [18], USA [[19], [20], [21]], Northern Ireland, Spain, and the Netherlands [22] and the UK [23]. All of them were conducted in vitro.

In three of the studies, samples were collected over periods of 4, 5, and 16 years, while the duration of sample collection in the other included studies was not indicated. The study population consisted of patients with cystic fibrosis. The microorganism we studied was BCC. The test environment was mentioned in one study, Microbank vials and Luria-Bertani agar. Bacterial cultures were stored at −80 °C in Microbank vials and were subcultured twice on Luria-Bertani agar before use. All cultures were incubated aerobically at 37 °C. They were performed using agar dilution method (two study), broth microdilution (two study), disc diffusion technique and genetic-based methods. Just in one of the studies, more than 100 *Burkholderia* species (ranging from 2 to 146) were isolated.

The MIC range utilized for combination of ceftazidime and Avibactam treatments is between <0.5 - 128 mg/L. In five studies, the MIC50 was reported as 4 mg/L and the MIC90 ranged between 2.4 and 16 mg/L.

Ceftazidime is one of the drugs of choice for the treatment of BCC. But the results of this study suggest that there is a higher sensitivity to beta-lactamase-ceftazidime when used in combination with each other. In the study by Everaert (2016), drug combinations including avibactam, ceftazidime were used. It should be noted that the combination of ceftazidime with avibactam causes at least 4-fold MIC-decrease compared to BCC species [13]. In the study by Massip (2018), the drug combination ceftazidime/avibactam was used. They had the best in vitro activity against BCC strains. When used alone, ceftazidime may require a dosage range of more than 256 mg/L to effectively treat certain BCC strains. However, when combined with avibactam, the therapeutic dose can be reduced to a range of 6–256 mg/L [14]. On average, the minimum inhibitory concentration (MIC) 50 of ceftazidime alone is twice as high as the combination drug [14,19]. Also, in the study by Caverly (2019), the drug combinations of ceftazidime-avibactam were used. Of course, ceftazidime/avibactam offer therapeutic options for managing airway infections due to opportunistic respiratory pathogens in persons with CF. However, the therapeutic efficacy of ceftazidime alone (91 %) is only equivalent to that of ceftazidime-avibactam in combination (97 %) [15]. In the study by Van-Dalem (2018), the drug combinations of ceftazidime, temocillin, piperacillin-tazobactam, and meropenem, at least 50 % of the strains were susceptible. However, among the mentioned drugs, ceftazidime-avibactam were more sensitive to BCC strains (81 %). This means that adding avibactam to ceftazidime increases sensitivity by 20 % [16].

In the study by Krisztina M (2017) and Gartner (2018), the drug combination ceftazidime-avibactam showed more sensitivity to BCC [18,19]. It should be noted that ceftazidime alone is susceptible to BCC strains, but the effect of sequential use of ceftazidime-avibactam is much less [18]. Also, In the study by Schaumburg (2022), The susceptibility rate for the ceftazidime-avibactam and ceftazidime compared with other drugs (trimethoprim/sulfamethoxazole) was detected second. on the other hand, Aztreonam in combination with ceftazidime/avibactam had no synergistic effect in our BCC isolates [17].

However, Mushtaq (2010) reported varying effects of beta-lactamases in relation to BCC when used with ceftazidime. She/he showed that NXL104 reduced the ceftazidime MIC by at least 4-fold in 35 cases and by ≥ 32-fold in 10 [23]. It is important to note that Dalem (2018) mentioned that more studies are needed to determine the sensitivity of ceftazidime and Avibactam on BCC. The dosage used was only reported in one study [16].

The combination of ceftazidime and Avibactam has been found to have a sensitivity rate of 67–97 % in cases of BCC, while resistance to ceftazidime was observed in 9–37 % of cases. however, this finding can be seen in Schaumburg study. He mentions the sensitivity of ceftazidime and ceftazidime-avibactam to BCC strains, was 53 % and 78 %, respectively [17].

Resistance to ceftazidime-avibactam can occur through various mechanisms, including reduced membrane permeability, altered penicillin-binding proteins (PBPs), and the presence of efflux pumps [13]. Additionally, specific enzymes such as PenA [16,19], PenB, and AmpC β-lactamases may contribute to resistance [16]. It is important to note that the effectiveness of ceftazidime-avibactam against *P. aeruginosa* isolates can vary depending on the specific combination of resistance determinants present [23] (Table 1).

**Table 1:** Basic characteristics of the included study.

First author	Country	Type of the study	Duration	Type of microorganism	Main outcome	Number of isolates	Agent	AST	MIC Range (mg/L)	MIC50 (mg/L)	MIC90 (mg/L)	Percentage susceptible, %	Resistance to CAZ	Resistance to other drugs
Mushtaq (2010) [23]	UK	in vitro	_	*P. aeruginosa*, BCC, and *A. baumannii*	It has variable ability to potentiate CAZ against *P. aeruginosa* and BCC isolates from patients with CF, this variation presumably reflecting the relative contributions of β-lactamases vis a' vis efflux in individual isolates	BCC (n = 54); *B. cenocepacia* (n = 6)	CAZ + NXL104	Agar dilution method	≤1–128 ≤	_^a^	_	36 of 54 (67 %) were susceptible to CAZ + NXL104 8 + 4 mg/L versus 5 of 54 (9 %) susceptible to CAZ alone	49 were resistant to CAZ, with MICs 0.8 mg/L, and 32 were highly resistant, with MICs 0.32 mg/L	CAZ MICs for the isolates varying in efflux-mediated resistance ranged from 0.03 to 16 mg/L, and were correlated with those for CBN, PIP, TZP and MEM, which are also excreted by MexA-OprM and other efflux systems
Everaert (2016) [13]	USA-Australia	in vitro	–	Burkholderia cepacia (*B. cepacia* LMG 1222, *B. multivorans* LMG 18822, *B. cenocepacia* LMG 16656, *B. vietnamiensis* LMG 10929, *B. ambifaria* LMG 19182 and *B. lata* LMG 6992), *P. aeruginosa* ATCC27853, *E. coli* ATCC 25922,	Addition of SUL, TAZ or AVI to CAZ, AMX, FOX, FEP or ATM leads to increased susceptibility (at least 4-fold MIC-decrease) in some BCC strains	–	CAZ:0.25 − 128 mg/L. AVI:4 mg/L	Microbank vials and Luria-Bertani agar	CAZ without AVI: 32–64 CAZ with AVI: 32-64	–	–	–	–	–
Krisztina M(2017) [19]	USA	in vitro	–	*B. multivorans*	CZA may serve as an alternative therapy for individuals with CF that develop *Burkholderia* spp. infections. When AVI is combined with CAZ, susceptibility to CAZ in MDR and XDR clinical strains of *Burkholderia* spp. isolated from CF respiratory specimens is restored.	146	CZA	Agar dilution method	0.5 - 64	4	8	93 % (n = 136)	n = 52 (35.5 %)	TOB (n = 140); IMP (n = 128); CIP (n = 119); MNO (n = 83); SXT (n = 54)
Dalem(2018) [16]	Belgian	in vitro	2012–2016	BCC strains	81 % of all BCC strains were susceptible to CZA. For TEM, CAZ, TZP, and MEM, at least 50 % of the strains were susceptible.	91	CZA	–	≤0.06 - 64 ≤	4	16	81 % (n = 74)	37 % (n = 34)	COL, n = 91; CIP and TGC, n = 90; AMK and TOB, n = 88); FEP, n = 57; ATM, n = 50; MEM, n = 45; PIP, n = 48; TZP, n = 38; TEM, n = 30; CT, n = 34; SXT, n = 16
Massip (2018) [14]	France	in vitro	–	*Burkholderia*, *Achromobacter*, *Cupriavidus*, *Inquilinus*, *Pandoraea* and *Ralstonia* clinical strains	Addition AVI increased CAZ activity against *Burkholderia* (88 % of strains were susceptible), CZA and MEM had the best in vitro activity against BCC strains.	10	CAZ and CZA	Broth microdilution method	CAZ:2–16, and CZA: 1–8	CAZ: 4 and CZA:4	CAZ:8 and CZA: 8	CAZ:50 (<=4) CZA:100 (<=8)	*B. cepacia*:10 (>8)	ATM:90 (>8); CT: 30 (>4); MEM: 0 (>8); TZP: 20 (>16); TEM: 0 (>16)
Gartner (2018) [18]	Spain	in vitro	–	All strain of BCC	CZA is the antibiotic that demonstrates the highest in vitro activity. The combination of AVI with CAZ restored susceptibility to CAZ in 60 % of the strains studied	17	–	Disc diffusion technique	–	4	24	–	–	–
Caverly (2019) [15]	USA- Canada	in vitro	2013–2018	*Burkholderia*, *Achromobacter*, *Stenotrophomonas*, and *Pandoraea* strains	CZA offers therapeutic options for managing airway infections due to opportunistic respiratory pathogens in persons with CF	20	CZA	genetic-based methods	for BCC (≤0.5 - >32); for *B. gladioli* (2 - >32)	for BCC = 4; for *B. gladioli* = 16	for BCC = 4; for *B. gladioli* = 16	for BCC = 97 %	for BCC = 9 % (n = 13);	for BCC, MEM = 15, CT = 16, MVB = 4, TZP = 22; for *B. gladioli*, MEM = 0, CT = 44, MVB = 0, TZP = 0
Schaumburg(2022) [17]	German	in vitro	2004–2020	BCC	The susceptibility rate for CZA was (78 %)	64	CZA	Broth microdilution	–	–	–	–	–	–
Tunney (2024)(22)	Northern Ireland, Spain and the Netherlands	in vitro	–	Burkholderia spp, Stenotrophomonas spp, Achromobacter spp, Pandoraea spp, Ralstonia	TGC and ERV demonstrate the lowest MIC50 and MIC90 against Burkholderia spp.	*Burkholderia* spp. (n = 106)	CZA	–	<0.25–16 <	4	16	–	–	–

**Abbreviation:** AST: Antimicrobial susceptibility method MIC: Minimum inhibitory concentration; BCC: *Burkholderia cepacia* complex; CAZ: Ceftazidime; SUL: sulbactam; TAZ: tazobactam; AVI: avibactam; MVB: meropenem/vaborbactam; IMR: imipenem/relebactam; CZA: ceftazidime/avibactam; CT: ceftolozane/tazobactam; AMX: amoxicillin; FOX: cefoxitin; FEP: cefepime; ATM: aztreonam; CF: Cystic fibrosis; MDR: multi-drug resistant; XDR: extensively-drug resistant; CBN: carbenicillin; PIP: piperacillin; TZP: piperacillin + tazobactam; TEM: temocillin; CIP: ciprofloxacin; COL: colistin; AMK: amikacin; TOB: tobramycin; ERV: eravacycline; FTB: cefepime/taniborbactam; TET: tetracycline; AMG: aminoglycoside; MEM: meropenem; TGC: tigecycline; LVX: levofloxacin, MI: minocycline; SXT: trimethoprim/sulfamethoxazole; MHA: Muller-Hinton agar.

a Not recorded.

## 4 Discussion

In this study, we review the in vitro susceptibility of ceftazidime-avibactam BCC isolates. The main finding of our review is that for the treatment of BCC diseases with resistant strains, one of the best options is ceftazidime-avibactam that in vitro activity can be an effective treatment option for infections. The BCC are opportunistic pathogens causing pneumonia in patients with cystic fibrosis (CF) and chronic granulomatous disease [24]. The clinical manifestations of BCC vary widely, ranging from no symptoms to severe respiratory infections and septicemia, particularly in patients with cystic fibrosis or chronic granulomatous illness [25].

Pathogens that are extremely resistant to drugs, like as BCC, significantly restrict the available therapy choices [26]. BCC infections can be treated empirically with trimethoprim-sulfamethoxazole, ceftazidime, meropenem, and doripenem [27]. However, the widespread use of these broad-spectrum antibiotics has led to a rise in multidrug-resistant BCC strains, decreasing treatment efficiency [28]. The in vitro resistance rate of BCC to ceftazidime has been reported at 30–40 % [[29], [30], [31], [32]]. Several studies have recommended against treating BCC infections with the ceftazidime regimen alone [33,34].

Our systematic review has established combination therapy by ceftazidime-avibactam is more effective for treatment of BCC infections. The ceftazidime-avibactam combination was shown to have the greatest in vitro susceptibility, which was 81 %, according to the findings of Krisztina M. et al. and Shazad Mushtaq Avibactam 20 % increased ceftazidime susceptibility [35,36]. Van Dalem A et al. has been shown that *B. cepacia* has a high sensitivity to ceftazidime-avibactam and trimethoprim-sulfamethoxazole, although it is resistant to fluoroquinolones and penicillin [2].

Tamma et al. reported the first instance of effective treatment of BCC bacteremia with ceftazidime-avibactam. [37]. Also, Papp-Wallace et al. reported raising susceptibility of 90 % of BCC and *B. gladioli* isolates to ceftazidime when combined with avibactam [19]. According to the findings of Massip et al.1, ceftazidime/avibactam and meropenem had the highest level of in vitro activity against BCC [8].

Later β-lactamase inhibitor, vaborbactam as a third generation β-lactamase inhibitor, in combination with meropenem has been compared with ceftazidime/avibactam by Caverly et al. [15]. Remarkably, meropenem/vaborbactam exhibited slightly better in vitro activity against BCC isolates compared to ceftazidime/avibactam, with MIC_90_ values of 2 μg/ml and 4 μg/ml, respectively. However, ceftazidime/avibactam has been suggested as a more appropriate option for treating BCC infections; however, several studies have indicated resistance to ceftazidime/avibactam. Only 47 % of *Burkholderia* strains were susceptible to ceftazidime, whereas the addition of the β-lactamase inhibitor avibactam significantly increased ceftazidime activity against *Burkholderia*, with 88 % of strains being susceptible [2,26,38,39].

In addition, there are different mechanisms of resistance to ceftazidime avibactam. For example, it is well known that the BCC is capable of producing an efficient outer penetration barrier that is comprised of restrictive porin proteins, effective efflux pumps, and beta-lactamases. This barrier inhibits the effectiveness of routinely used antibiotics and correlates with resistance to quinolones, coumarins, cyclothialidines, and pyrimethamine The else often high-level acquired or intrinsic resistance of non-enteric bacteria such as *P. aeruginosa* and Burkholderia species is in no small part attributable to synergy between reduced penetration into and efflux from the cell [[40], [41], [42]].

Furthermore, β-Lactamases are enzymes responsible for the hydrolysis of β-lactam antibiotics, rendering them non-functional and impeding their way to the penicillin-binding protein located in bacterial cell membranes. ceftazidime, a β-lactam antibiotic, and avibactam, a β-lactamase inhibit. Also, BCC produces two known chromosomal β-lactamases, consisting of a Pen-like family class A mutant β-lactamase and an AmpC class C β-lactamase [7,17,43]. Therefore, combination of β-Lactams with β-Lactamase inhibitors has been introduced as new approach combatting infections causes MDR gram-negative bacilli, like BCC [44,45].

It has been shown that some incidences of ceftazidime-avibactam resistance are caused by mutations in the *bla*KPC-3 gene that is carried by plasmids. In a fascinating discovery, it was shown that some mutations in *bla*KPC-3, which conferred resistance to ceftazidime-avibactam, were linked to reductions in (MICs) for carbapenems and other β-lactam antibiotics [46,47].

ESBL-positive isolates (99.9 % susceptible; MIC_90_, 0.5 μg/ml), AmpC-positive isolates (100 % susceptible; MIC_90_, 0.5 μg/ml), and isolates positive for both an ESBL and an AmpC β-lactamase (100 % susceptible; MIC_90_, 1 μg/ml) were almost uniformly susceptible to ceftazidime-avibactam [48].

The first generation of β-Lactamase inhibitors (e.g. clavulanic acid, sulbactam, and tazobactam) were not more effective against BCC [49]. For example, PenA1 produced by *B. multivorans* can hydrolyze clavulanic acid, sulbactam, and tazobactam; strains that produce blapenA1 are not vulnerable to these well-known combinations of β-lactam and β-lactamase inhibitors [50].

Ceftazidime in combination with avibactam was approved by the U.S. Food and Drug Administration in 2015 as a novel cephalosporin-diazabicyclooctane β-lactamase inhibitor. The MIC of ceftazidime was found to be reduced by a range of four to thirty-two fold, as demonstrated by the researches that were evaluated [13,23]. The addition of avibactam allowed for a decrease in the MIC_50_ and the MIC_90_ of ceftazidime of more than four and two 2-fold dilution, respectively [51]. In other words, the addition of avibactam significantly enhanced the activity of ceftazidime, resulting in susceptibility rates comparable to those of meropenem [8]. Similarly, *B. cenocepacia* exhibited the highest in vitro susceptibility to ceftazidime-avibactam, at 88 % [52,53].

Lahiri and coworkers have identified residues in AmpC variants with the ability to generate resistance to avibactam, although the authors suggest that the ability of avibactam to mimic the key interaction of a β-lactam substrate combined with its tight binding likely confer a barrier to the development of clinical resistance [54]. Hence, a new strategy, such as replacing ceftazidime with other β-lactams, is essential for managing BCC infections. This has been assessed by Zeiser et al., that investigated the susceptibility of BCC isolates from CF patients to piperacillin/avibactam. They found that 13 out of 14 ceftazidime/avibactam resistant BCC isolates were susceptible to piperacillin/avibactam. However, piperacillin/avibactam has not been approved for clinical use yet [14,16]. It is necessary to conduct further studies assessing the potency of novel β-lactamase inhibitors in combination with β-lactams against BCC species separately.

Our findings could be of use to physicians in the process of administering antibiotics to their patients. On the other hand, there is a need for more susceptibility studies for BCC species, as well as clinical research to evaluate the clinical results.

## 5 Conclusion

Ceftazidime-avibactam, a novel combination antibiotic, has recently emerged as a potential therapeutic option against *B. cepacia* opportunistic pathogens. Our systematic search with 8 articles showed that *B. cepacia* is more sensitive to this new combination therapy feature.

The exploration of ceftazidime-avibactam's susceptibility against *B. cepacia* marks a promising avenue in the ongoing battle against antibiotic resistance. While challenges exist and some of BCC isolates exhibited resistance to ceftazidime-avibactam, the potential benefits underscore the importance of further research and clinical trials to establish the role of ceftazidime-avibactam in the treatment paradigm for BCC infections. This pursuit is essential in addressing the pressing need for effective therapies against multidrug-resistant pathogens, offering hope for improved outcomes in the management of BCC infections.

## CRediT authorship contribution statement

**Abolfazl Rafati Zomorodi:** Writing – original draft, Visualization, Validation, Investigation, Data curation, Conceptualization. **Leila Ghanbari_Afra:** Writing – original draft, Visualization, Validation, Investigation, Data curation, Conceptualization. **Hossein Faridafshar:** Writing – original draft, Visualization, Validation, Investigation, Data curation, Conceptualization. **Seyed Nooreddin Faraji:** Writing – original draft, Methodology, Investigation. **Fatemeh Zarepour:** Writing – original draft, Methodology, Investigation. **Maryamosadat Mavaei:** Writing – original draft, Methodology, Investigation. **Mohammad Rahmanian:** Writing – review & editing, Validation, Project administration.

## Ethics approval and consent to participate

Not applicable.

## Consent for publication

Not applicable.

## Availability of data and materials

No data was used for the research described in the article.

## Fundings

This research did not receive any specific grant from any funding agency in the public, commercial, or not-for-profit sectors.

## Declaration of competing interest

Please find enclosed a manuscript entitled “**In Vitro Susceptibility of *Burkholderia cepacia* to Ceftazidime-Avibactam: a systematic review**” which I would like to submit to your consideration for eventual publication in your esteemed journal. On behalf of the co-authors and myself, the present work is original and has not been published, nor is it being considered for publication in another journal. All authors know of no conflicts of interest associated with this publication, and there has been no significant financial support for this work that could have influenced its outcome.
